# Efficacy of *careHPV*™ human papillomavirus screening versus conventional cytology tests for the detection of precancerous and cancerous cervical lesions among women living with HIV‐1 in Lao People's Democratic Republic

**DOI:** 10.1002/cam4.4502

**Published:** 2022-03-08

**Authors:** Phimpha Paboriboune, Keokedthong Phongsavan, Phetsamone Arounlangsy, Bruno Flaissier, Oukham Aphayarath, Prasit Phimmasone, Ketmala Banchongphanith, Mixi Xayaovong, Gonzague Jourdain, Anne‐Marie Schott, Mitra Saadatian‐Elahi, Laurent Magaud, Amna Klich, Nicole Ngo‐Giang‐Huong, Isabelle Heard, Muriel Rabilloud, Valentina Sanchez Picot, Christophe Longuet

**Affiliations:** ^1^ Centre d'Infectiologie Christophe Mérieux (CICML) Vientiane Lao‐PDR; ^2^ Setthatirath Hospital Vientiane Lao‐PDR; ^3^ Public Health Science University Vientiane Lao‐PDR; ^4^ Fondation Mérieux Phu Phanang National Bio‐Diversity Vientiane Lao‐PDR; ^5^ HIV Center Setthathirath Hospital Vientiane Lao‐PDR; ^6^ HIV Center Mahosot Hospital Vientiane Lao‐PDR; ^7^ National Center for HIV/AIDS and STI Vientiane Lao‐PDR; ^8^ French National Research Institute for Sustainable Development (IRD) Marseille France; ^9^ Université Claude Bernard Lyon 1 Villeurbanne France; ^10^ Service Hygiène Epidémiologie Infectiovigilance et Prévention Centre Hospitalier Edouard Herriot Hospices Civils de Lyon Lyon France; ^11^ Public Health, Epidemiology and Evolutionary Ecology of Infectious Diseases (PHE3ID) – Inserm – U1111 – UCBL Lyon 1 – CNRS – UMR5308 – ENS de Lyon Lyon France; ^12^ Hospices Civils de Lyon Pole Santé Publique Lyon France; ^13^ Université de Lyon Lyon France; ^14^ Université Lyon 1 Villeurbanne France; ^15^ Service de Biostatistique‐Bioinformatique Pôle Santé Publique Hospices Civils de Lyon Lyon France; ^16^ Équipe Biostatistique‐Santé Laboratoire de Biométrie et Biologie Évolutive CNRS UMR 5558 Villeurbanne France; ^17^ French National Human papillomavirus Reference Laboratory Institut Pasteur Paris France; ^18^ Fondation Mérieux Lyon France

**Keywords:** *careHPV*™, cervical cancer, HIV‐1, Lao PDR, liquid‐based cytology, Papanicolaou test

## Abstract

**Background:**

In the Lao People's Democratic Republic (Lao PDR), cervical cancer is the third leading cause of women cancer.

**Aims:**

The objective of this cross‐sectional study was to compare the efficacy of *careHPV*™ test versus conventional Pap smear or Siriraj liquid‐based cytology in the detection of cervical cancer in women living with human immunodeficiency virus type 1 (HIV‐1).

**Materials & Methods:**

Overall, 631 women consented to participate. Four cervical specimens were taken for the purpose of conventional Pap smear, Siriraj liquid‐based cytology, *careHPV*™ test, and HPV‐16 genotyping. The exact McNemar test was used to compare the efficacy and diagnostic performance of the tests.

**Results:**

Of the 631 women with follow‐up, 331 were human papillomavirus (HPV) negative. High‐grade squamous intraepithelial lesions were found in 37 women, biopsy‐proven high‐grade cervical intraepithelial neoplasia in 50 women, and invasive carcinoma in seven women. The proportion of women with high‐grade cervical lesion or carcinoma detected after abnormal *careHPV*™ test was higher (6.02%; 95% confidence interval [CI]: 4.4–8.1) than that detected by conventional Pap smear (4.59%; 95% CI: 3.2–6.5). *careHPV*™ and HPV‐16 genotyping had, respectively, the highest sensitivity (80.8%; 95% CI: 67.4–89.5) and specificity (92.2%; 95% CI: 89.8–94.2). HPV‐16 was the most frequently detected genotype.

**Conclusions:**

*careHPV*™ test represents a screening option in Lao PDR, particularly in women living with HIV‐1 because of higher prevalence of chronic HPV in this population.

## Graphical abstract

LaoCol study provided health authorities in Lao People's Democratic Republic with a basis for improving their national cervical cancer screening policy and some epidemiological information useful for the establishment of a possible human papillomavirus vaccine prevention policy.

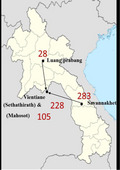

## 1 INTRODUCTION

With more than 500,000 new cases in 2018, cervical cancer is the fourth most frequent cancer among women worldwide. Overall, 70% of cervical cancers are due to persistent infection with human papillomavirus (HPV) oncogenic genotypes 16 and 18.^1^ There is a high disparity in cervical cancer incidence and mortality between developed and developing countries. Based on GLOBOCAN estimates in 2018, the incidence of cervical cancer in the developed world was 10 cases per 100,000 compared to 18 cases per 100,000 populations in the developing world.^2^ In the Lao People's Democratic Republic (Lao PDR), cervical cancer is the third leading cause of women cancer with an estimated annual number of 320 cases and an estimated crude annual incidence rate of 9.2 per 100,000 women.^3^

To prevent cervical cancer, the World Health Organization (WHO) provided recommendations for a screen‐and‐treat program.^4^ In countries with no cervical cancer prevention and control program, the WHO strategies to be implemented are based on available resources.^4^ Among the five different strategies proposed, one is based on visual inspection of the cervix with 3%–5% acetic acid and another one consists of conventional cytology. HPV testing relies on different molecular techniques that require specific infrastructure and remains expensive. A simple and affordable DNA test, the *careHPV*™ test, based on signal amplification of HPV DNA, allows the detection of 14 high‐risk genital HPV (HR‐HPV) genotypes and presents several advantages over the standard molecular techniques^5^: it does not require sophisticated molecular laboratory infrastructure and can be performed without electricity or running water. In addition, the results are obtained within 2–5 h. In a cross‐sectional study carried out in China, the sensitivity and specificity of digene high‐risk HPV HC2 DNA test for cervical specimens were 97.1% (95% confidence interval [CI]: 93.2–100) and 85.6% (95% CI: 84.2–87.1), respectively (areas under the curve were not significantly different from the *careHPV*™ test on cervical specimens, *p* = 0.0163).^5^

Infection with human immunodeficiency virus type 1 (HIV‐1) increases the risk of developing precancerous and cancerous cervical lesions.^6^ In the USA, the standardized incidence ratio for cervical cancer in women living with HIV‐1 (1996–2012) was 3.24 (95% CI: 2.94–3.56).^7^ As compared to otherwise healthy women, those living with HIV‐1 have also higher prevalence of HR‐HPV infection and related diseases. The impact of combination antiretroviral therapy (cART) on HR‐HPV–related lesions has been investigated in a recent meta‐analysis that found a significantly lower prevalence of HR‐HPV in treated women (adjusted odds ratio [aOR] = 0.83, 95% CI: 0.70–0.99), a decreased risk of precancerous lesions (aOR = 0.59, 95% CI: 0.40–0.87), and a reduction in invasive cervical cancer incidence after adjustment for CD4 cell count and treatment duration (crude HR = 0.40, 95% CI: 0.18–0.87).^8^

In Lao PDR, the prevalence of HIV‐1 infection is relatively low, around 0.2% in 15–49 years old.^3^ In 2011, the number of women living with HIV‐1 was estimated at 5,263, half of them receiving cART under the National HIV/AIDS Program supported by the Global Fund.^9^ The estimates of cervical cancer among women living with HIV‐1 are not known.

Regular screening is considered as a key tool in the prevention of cervical cancer, in particular in women living with HIV‐1 for whom the primary prevention by vaccination is not effective.^10^ According to clinical guidelines, women living with HIV‐1 should be screened for cervical cancer twice within the first year following HIV diagnosis, and annually thereafter if the results are normal.^11^ However, screening and/or preventive vaccination programs against HPV infection does not exist in Lao PDR. The screening coverage for women aged 18–69 years was estimated at 2.2%, 5.2% in urban areas and 1.4% in rural areas of Lao PDR.^12^ In addition to this low coverage, most women do not know that HPV causes cervical cancer.^9^

Attending HIV treatment centers could be an opportunity to screen for cervical cancer. The most successful strategy for cervical cancer screening, the conventional Pap smear test, that has shown to help reducing the cervical cancer rates by 60%–90% in developed countries, relies on a level of infrastructure unattainable in most of the developing world. In the past few years, several tests looking for the DNA of HR‐HPV have demonstrated high sensitivity in the detection of cervical intraepithelial neoplasia (CIN).^13^, ^14^ Women living with HIV‐1 in Lao PDR could benefit from such tests for an early detection of cervical cancer. The LaoCol study was initiated to assess the efficacy of available screening tests and to inform the Laotian healthcare authorities on the importance of screening programs among women living with HIV‐1.

## 2 OBJECTIVES

The main objective of this cross‐sectional study was to compare the efficacy of *careHPV*™ test versus conventional Pap smear or Siriraj liquid‐based cytology in the detection of histology proven high‐grade CIN or invasive cervical cancer in a cohort of women living with HIV‐1 in Lao PDR.

The secondary objectives were (1) to estimate and compare the performances (sensitivity, specificity, positive, and negative predictive values) of these three tests in the detection of histology proven high‐grade CIN or invasive cancer (CIN2+), (2) to assess the agreement between the two cytological tests and between *careHPV*™ and PapilloCheck HPV test, (3) to determine the prevalence of different types of oncogenic HPV strains among women living with HIV‐1, and (4) to identify factors associated with CIN grade 2 and plus (CIN2+).

## 3 METHODS

### 3.1 Study design and participants

The study population included women living with HIV‐1 who attended four HIV reference centers in Lao PDR in Vientiane (Mahosot, Setthathirath), Savannakhet and Luang Prabang from January 2014 to May 2015 and who consented to participate.

Eligible women had documented HIV‐1 positive status, were aged between 25 and 65 years, and had no history of cervical cancer, pelvic radiotherapy, or recent pregnancy (until 3 months after delivery). The study protocol and consent forms were approved by the Lao PDR Ethical Review Committee (National Ethical Committee for Health Research, September 13th, 2013) and the Thailand Ethical committee (Ethic Committee, Faculty of Associated Medical Science, Chiang Mai University, January 20th, 2014). All participants provided written informed consent.

Previous medical history, the most recent CD4 cell count, HIV viral load, and current cART medication were collected from the patients' medical record.

Gynecological examination was performed in all participants by the same gynecologist. Four cervical specimens were collected from each patient: (1) with a spatula for conventional pap smear cytology; (2) with a cytobrush placed into a plastic vial containing a specific preservative solution for Siriraj liquid‐based cytology; (3) with a cytobrush placed into *careHPV*™ collection medium for HPV testing using *careHPV*™ (QIAGEN); and (4) with a cytobrush placed into PapilloCheck^®^ collection medium for HPV genotyping using PapilloCheck^®^. Physical examination included a systematic colposcopy; acetowhite areas were biopsied and processed for histological examination. Patients with positive HR‐HPV DNA and negative colposcopy were proposed a gynecologic follow‐up 6 months later.

Appropriate free of charge treatment was provided to patients identified with CIN lesions or cancer.

### 3.2 Procedures

#### 3.2.1 Reference method

Systematic colposcopy and histological examination of biopsies carried out in women with abnormal colposcopy were considered as the reference method to determine the presence of high‐grade cervical lesions or cancer. Absence of high‐grade lesions was defined as normal colposcopy or absence of high‐grade lesions (CIN2 and CIN3) and carcinoma by histological examination.

In patients with normal colposcopy and one abnormal cytological test (conventional Pap smear or Siriraj liquid‐based cytology), a second colposcopy was performed to re‐evaluate the presence of high‐grade cervical lesions or cancer.

#### 3.2.2 Conventional Pap smear or Siriraj liquid‐based cytology

Both cervical cytology tests were evaluated, regardless of the HPV results. The results were categorized according to the 2001 Bethesda System terminology^15^: negative; atypical squamous cells of undetermined significance (ASC‐US); low‐grade squamous intraepithelial lesion (LSIL); high‐grade squamous intraepithelial lesion (HSIL); atypical squamous cells, cannot exclude HSIL (ASC‐H); and cancer. A positive cytology test was defined as the presence of any cytological abnormalities.^16^

#### 3.2.3 HPV DNA testing and genotyping

Specimens collected for HPV DNA testing were stored at room temperature and sent to the Centre d’Infectiologie Christophe Mérieux (CICML in Vientiane, Lao PDR) High‐risk HPV detection was performed within 14 days after sampling using the *care*HPV™ test (Qiagen), a signal‐amplification diagnostic test allowing the detection of 14 HR‐HPV genotypes (16, 18, 31, 33, 35, 39, 45, 51, 52, 56, 58, 59, 66, and 68).^6^

##### Genotyping

Specimens collected for the identification of specific HPV genotypes were kept at −20°C and sent by batches to the virology laboratory at Chiang Mai University on a monthly basis. HPV DNA genotyping was performed using the PapilloCheck^®^ test system (Greiner BioOne GmbH, Frickenhausen, Germany). The test can identify 24 HPV genotypes including HPV6, 11, 16, 18, 31, 33, 35, 39, 40, 42, 43, 44/55, 45, 51, 52, 53, 56, 58, 59, 66, 68, 70, 73 and 82.^17^

#### 3.2.4 Sample size

The sample size was estimated to compare the efficacy of two screening tests, measured by the proportion of women detected with a high‐grade lesion (CIN2+) among the screened women. We assumed an expected difference of 1.7% in the proportion of detected women between the two screening tests (2.8% with conventional Pap smear and 4.5% with *careHPV*™), and an expected proportion of discordant pairs of 2.7%. Based on these parameters, the requested sample size to detect a significant difference with a unilateral alpha risk of 5% and power of 80% was 600 patients (calculated with b Query version 5.0).

#### 3.2.5 Statistical analysis

Quantitative characteristics were described as median and interquartile range and qualitative characteristics as absolute and relative frequencies in each category. The proportions of screened women with high‐grade cervical lesion or carcinoma detected by the different screening tests were compared by the exact McNemar test. Sensitivity, specificity, positive predictive value, negative predictive value (NPV), positive likelihood ratio (LR+), negative likelihood ratio (LR−), and their 95% confidence interval (CI) were estimated based on the Wilson Score method. The level of agreement for cytology interpretation between conventional Pap smear and Siriraj liquid‐based cytology was estimated after classification into two categories: negative or minor cytological abnormalities (negative, ASC‐US, or LSIL) versus significant cytological abnormalities (ASC‐H, HSIL, or invasive cancer). The agreement between the *care*HPV™ test and the PapilloCheck^®^ HPV assay was estimated at aggregated level for the 14 high‐risk HPV genotypes detected by the *care*HPV™ test. Both agreements were measured using the Cohen kappa coefficient with 95% CI. To identify factors associated with CIN2+ histology, univariate logistic regressions were performed using age, last known CD^4+^, nadir CD^4+^ Lymphocytes count, and HPV test results in the model. Two multivariate logistic regressions were carried out including the above‐mentioned characteristics and *careHPV*™ or HPV‐16 results in the first and second models, respectively. The effect of each factor was estimated by an odds ratio and its 95% CI.

All statistical analyses were carried out using the R software, version 3.3.1 (Free Software Foundation, http://www.r‐project.org).

## 4 RESULTS

From January 31, 2014 to May 27, 2015, a total of 772 women consented to participate. In total, 128 women did not attend the gynecology consultation, one women was diagnosed with invasive cervical cancer and sent for appropriate care without HPV test, and 12 women were excluded due to unsatisfactory biopsy results, leaving 631 women for analysis (Figure S1).

### 4.1 Characteristics of the study population

Table 1 summarizes demographic and behavioral characteristics of the study population. With a median age of 36 years (interquartile range, IQR, 31.1–42.4), 60% of the women were engaged in a relationship. Median age at first sexual experience was 19 years and 94% reported less than 4 lifetime sexual partners. Median age at HIV diagnosis was 31 years (IQR: 27–37) and median nadir CD^4+^ was 151 (IQR: 50–274) cells/mm^3^. At inclusion, 94.2% of women were on cART, with a median CD^4+^ cell count of 374 (IQR: 240–504) cells/mm^3^ and median HIV load of 4341 copies/ml (IQR: <250–1,094,000). Of the 501 women with known results, 471 (94%) had HIV load <200 copies/ml.

**TABLE 1:** Characteristics of the study population

Characteristics	All patients *N* = 631
Age, years, median (IQR)	36.0 (31.1–42.4)
Marital status	
Married/cohabitation	379 (60.1)
Separated/divorced/single	143 (22.6)
Widowed	109 (17.3)
Number of children, median (IQR)	1 (1–2)
Age at first sexual encounter, years, median (IQR)	19 (18–22)
Level of education, *n* (%)	
Illiterate	74 (11.7)
Primary	256 (40.6)
Secondary	264 (41.8)
Technical/University	37 (5.9)
Lifetime number of sexual partners, *n* (%), *N* = 629	
1–3	596 (94.8)
>=4	33 (5.2)
Characteristics of HIV infection	
Age at HIV diagnosis, years, median (IQR)	31 (27–37)
Prior diagnosis of AIDS (stage 3 or 4), *n* (%)	397 (60.1)
Current cART, *n* (%)	588 (93.2)
CD^4+^, cell count /mm^3^, median (IQR), *N* = 624	374 (240–504)
Nadir CD^4+^, cell count/mm^3^, median (IQR), *N* = 626	151 (50–274)
HIV load, copies/ml, median (IQR), *N* = 501	4341 (<20–109,4000)
HIV viral load < 200 copies/ml, *n* (%), *N* = 501	471 (94)

Abbreviation: HIV, human immunodeficiency virus.

### 4.2 Description of tests and biopsy results

Overall, 631 women had a follow‐up, of whom 222 (35.2%) were HPV positive (Figure S2). At least one LR‐HPV and HR‐HPV genotype was found in 41 and 107 women, respectively. LSIL and HSIL were observed in 139 (30.6%) and 37 (5.8%) women, respectively. Biopsy‐proven high‐grade CIN was found in 51 women, with CIN2 in 22 cases and CIN3 in 18 cases. In situ and invasive carcinoma (IC) were detected in three and four women, respectively (Table 2). Conventional cytology was unable to detect any of the ICs.

**TABLE 2:** HPV status of women with biopsy‐proven high‐grade CIN

Biopsy/colposcopy	Gold CIN1/low grade	CIN2/high grade	CIN3/high grade	In situ carcinoma	Invasive carcinoma	Normal
HPV test						
Negative test	13 (25.5%)	5 (22.7%)	0 (0.0%)	0 (0.0%)	0 (0.0%)	308 (57.8%)
Positive test	38 (74.5%)	17 (77.3%)	18 (100.0%)	3 (100.0%)	4 (100.0%)	225 (42.2%)
Total	*N* = 51	*N* = 22	*N* = 18	*N* = 3	*N* = 4	*N* = 533

Abbreviations: CIN, cervical intraepithelial neoplasia; HPV, human papillomavirus.

### 4.3 Comparison of performances of conventional Pap smear or Siriraj liquid‐based cytology with *careHPV*™

The efficacy of *careHPV*™ test as compared to conventional Pap smear or Siriraj liquid‐based cytology in the detection of cervical cancer is shown in Table 3. Among screened women, the proportion of those with a high‐grade cervical lesion or a carcinoma detected after abnormal *careHPV*™ test was significantly higher (6.02%; 95% CI: 4.4–8.1, *n* = 38/631) than that detected by conventional Pap smear (4.59%; 95% CI: 3.2–6.5, *n* = 29/631, *p* = 0.05). The efficacy of *careHPV*™ versus Siriraj liquid‐based cytology and PapilloCheck versus *careHPV*™ was not significantly different.

**TABLE 3:** Efficacy of different techniques in the detection of cervical cancer

Test	Proportion of high‐grade lesion/carcinoma (95% CI)	*careHPV*™	Conventional Pap smear
		*p* value	*p* value
*careHPV*™	6.02 [4.4–8.1]	–	–
Conventional Pap smear	4.59 [3.2–6.5]	0.05	–
Siriraj liquid‐based cytology	5.86 [4.3–7.9]	1	0.06
PapilloCheck	6.66 [4.9–8.9]	0.12	0.002
PapilloCheck HPV‐16	1.90 [1.1–3.3]	<0.001	<0.001

Abbreviation: HPV, human papillomavirus.

Performance of different techniques to detect CIN2 or higher grade lesions is shown in Table 4A. *careHPV^TM^* had the highest sensitivity (80.8%, 95% CI: 67.4–89.5) while HPV‐16 genotyping showed the lowest sensitivity (25.2%, 95% CI: 15.2–39.5). The best specificity was seen for HPV‐16 genotyping (92.2%, 95% CI: 89.8–94.2). *care*HPV™ and liquid‐based cytology had similar ability to confirm and eliminate the presence of CIN2+ histology lesions with a LR+ of 2.6 and a LR− of 0.3. Sensitivity and specificity of each test stratified by age group are provided in Table S1. *care*HPV™ and Siriraj liquid‐based cytology showed 100% sensitivity in women more than 50 years old while the specificity did not vary between the age strata. The accuracy of each test in terms of the proportion of true positive and false negative is reported in Table 4B.

**TABLE 4A:** Performance of HPV testing, conventional Pap smear, or Siriraj liquid‐based cytology to detect CIN2 or worse histology in women living with HIV‐1

Test	CIN2 or worse Histology			Sensitivity (95% CI)	Specificity (95% CI)	PPV (95% CI)	NPV (95% CI)	LR+ (95% CI)	LR− (95% CI)
	Yes	No	Total						
*CareHPV* positive	38	184	222	80.8% (67.4–89.5)	68.5% (64.6–72.1)	17.1% (12.7–22.6)	97.8% (95.8–98.8)	2.6 (2.1–3.1)	0.3 (0.1–0.5)
*CareHPV* negative	9	400	409						
HPV‐16 positive	12	45	57	25.2% (15.2–39.5)	92.2% (89.8–94.2)	21.05% (12.4–33.2)	93.9% (91.6–95.5)	3 (1.8–5.8)	0.8 (0.7–0.9)
HPV‐16 negative	35	539	574						
ASC‐US or worse	29	201	230	61.7% (47.4–74.2)	65.5% (61.6–69.3)	12.6% (9–17.6)	95.5% (93–97.1)	1.8 (1.4–2.3)	0.6 (0.4–0.8)
Negative cytology (conventional Pap smear)	18	383	401						
ASC‐US or worse	37	180	414	78.7% (65–88)	69.2% (65.5–73)	17.1% (12.6–22.6)	97.6% (95.6–98.6)	2.6 (2.1–3.1)	0.3 (0.1–0.5)
Negative cytology (Siriraj liquid based)	10	404	217						

Abbreviations: ASC‐US, Atypical squamous cells of undetermined significance or low‐grade squamous intraepithelial lesion or high‐grade squamous intraepithelial lesion or invasive cervical cancer; HIV‐1, human immunodeficiency virus‐1; HPV, human papillomavirus; LR−, negative likelihood ratio; LR+, positive likelihood ratio; NPV, negative predictive value; PPV, positive predictive value.

**TABLE 4B:** Accuracy of HPV testing, conventional Pap smear, or Siriraj liquid‐based cytology to detect CIN2 or worse histology in women living with HIV‐1

Test	Accuracy (%)	95% CI
*careHPV*	69.4	[65.7–72.88]
Conventional Pap smear	65.2	[61.4–68.9]
Siriraj liquid‐based cytology	69.9	[66.2–73.3]

Note

Accuracy: Proportion of true positive and false negative.

Abbreviations: HIV‐1, human immunodeficiency virus‐1; HPV, human papillomavirus.

With cytology diagnoses classified into two categories: negative or minor cytological abnormalities (negative, ASC‐US, or LSIL) versus significant cytological abnormalities (ASC‐H and HSIL), the agreement between conventional Pap smear and Siriraj liquid‐based cytology was poor (Cohen kappa: 0.39, 95% CI: 0.21–0.52).

### 4.4 HPV genotyping virology

**FIGURE 1:**
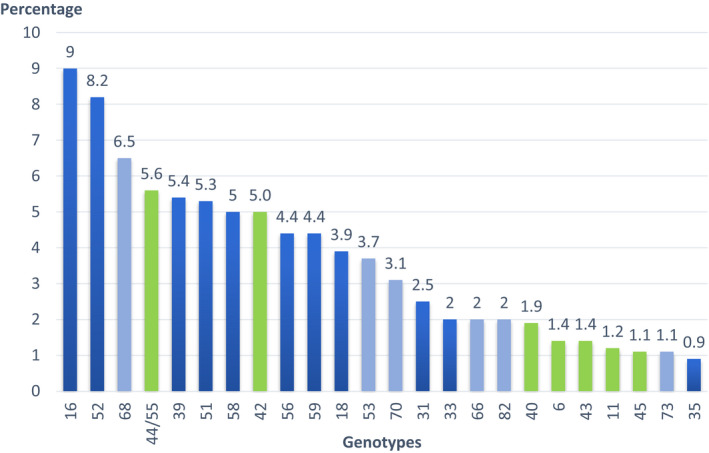
Human papillomavirus genotypes detected by PapilloCheck

HPV genotypes detected by PapilloCheck are presented in Figure 1. HPV‐16 was the most frequently detected genotype (9%), followed by HPV 52 (8.4%) and 68 (6.7%).

The agreement between *care*HPV™ and PapilloCheck HPV test assessed at aggregated level for 14 HPV high‐risk types in 591 specimens was moderate (Cohen kappa 0.7, 95% CI: 0.55–0.86) (Table S2).

### 4.5 Prevalence of cervical HPV infection and associated diseases

Factors associated with CIN2+ lesions in HIV‐1–infected women are summarized in Table 5. Positive *care*HPV™ test (OR: 9.39, 95% CI: 4.6–21.4) and presence of HPV‐16 (OR: 4.26, 95% CI: 1.9–8.7) were significantly associated with CIN2+ lesions. Multivariate analysis with HPV‐16 in the model showed that nadir Lymphocyte CD4 account less than 200 cells per mm^3^ was significantly associated with a decreased risk of CIN2+ in HIV‐1–infected patients (OR: 0.51, 95% CI: 0.3–0.9).

**TABLE 5:** Factors associated with the risk of CIN2+ in HIV‐infected women

	*n*	(%)	Logistic model								
			Univariable			Multivariable with *careHPV*			Multivariable with HPV16		
			OR	(95% CI)	*p*	OR	(95% CI)	*p*	OR	(95% CI)	*p*
Age at baseline											
18–39 years	399	30									
40–49 years	151	13	1.16	(0.6–2.2)	0.67	1.45	(0.7–2.9)	0.28	1.41	(0.7–2.8)	0.33
≥50 years	81	4	0.64	(0.2–1.7)	0.41	0.72	(0.2–2.0)	0.57	0.75	(0.2–2.0)	0.60
CD^4+^ T lymphocytes count (seven missing)											
≥300 cells/mm^3^	403	27									
<300 cells/mm^3^	221	20	1.39	(0.7–2.5)	0.29	1.11	(0.55–2.2)	0.75	1.70	(0.9–3.3)	0.11
Nadir CD^4+^ T lymphocytes count (five missing)											
≥200 cells/mm^3^	254	24									
<200 cells/mm^3^	372	23	0.63	(0.4–1.2)	0.13	0.57	(0.3–1.14)	0.11	0.51	(0.3–0.9)	0.04
Positive *CareHPV*											
No	409	9									
Yes	222	38	9.18	(4.5–20.6)	<0.0001	9.39	(4.6–21.4)	<0.0001	–	–	–
Cervical HPV‐16											
No	574	35									
Yes	57	12	4.11	(1.9–8.3)	0.0001	–	–	–	4.26	(1.9–8.7)	<0.0001
Cervical HPV52											
No	578	40									
Yes	53	7	2.05	(0.8–4.6)	0.10	–	–	–	–	–	–

Abbreviations: HIV, human immunodeficiency virus; HPV, human papillomavirus

## 5 DISCUSSION

The primary objective of this cross‐sectional, multicenter study was to compare the efficacy of *careHPV*™ test versus conventional Pap smear or Siriraj liquid‐based cytology in the detection of cervical cancer in a cohort of women living with HIV‐1 in Lao PDR. The results showed that the efficacy of *careHPV*™ test to detect high‐grade cervical lesions or carcinoma was significantly higher than the conventional Pap smear but was not significantly different than that of Siriraj liquid‐based cytology. However, as the careHPV™ molecular test does not have an internal control to assess the sampling error, we cannot consider samples negative for high‐risk HPV as true high‐risk HPV negative samples.

All women were examined and underwent colposcopy and biopsy by the same gynecologist. We found high prevalence of HR‐HPV (34.8%), similar to what has been reported in a study of women living with HIV‐1 in Thailand,^18^ emphasizing the potential benefits of integrating cervical screening program in this population. The conventional Pap smear cytology is the only method used throughout hospitals in Lao PDR,^19^ though this method may not be the gold standard for the diagnosis of cervical cancer.^9^

Cytology, either conventional Pap smear or Siriraj liquid‐based cytology, visual inspection with acid acetic and HPV testing are the current screening tests for cervical cancer and pre‐cancerous lesions, with large disparities in their access worldwide. Cervical cytology Pap smear test is the most common screening method, established in high‐income countries as the primary screening test, and has led to significant decrease in the incidence of and mortality from cervical cancer.^4^ However, this method seems to be less effective in developing countries,^4^ probably due to inadequate skill for good quality sample collection.^4^ Conventional Pap smear requires frequent visits and trained staff. The test can have false‐negative and false‐positive results due to inadequate sampling and slide preparation, errors in laboratory detection, and interpretation. The accuracy of this screening tool remains controversial with sensitivity and specificity ranging from 30% to 87% and from 86% to 100%, respectively.^20^

Siriraj liquid‐based cytology was developed to improve the diagnostic reliability of conventional Pap smear. Siriraj liquid‐based cytology rinses cervical cells in preservatives so that blood and other potentially obscuring material can be separated. The rate of detection for epithelial cell abnormalities was similar using either conventional Pap smear or Siriraj liquid‐based cytology in some studies^1^, ^21^, ^22^ while others reported better performance of the Siriraj liquid‐based cytology.^23^, ^24^ In our study, Siriraj liquid‐based cytology was more sensitive for the detection of HSIL than the conventional Pap smear.

Molecular tests for the detection of HPV DNA can increase the sensitivity to detect CIN2+ lesions, thereby improving the prevention of cervical cancer. Different molecular assays for HR‐HPV detection in primary screening for cervical cancer prevention have provided a significant increase in sensitivity and reproducibility compared to the conventional Pap smear test.^25^ HPV‐based screening using affordable and rapid tests such as *careHPV*™ for a single‐visit screen‐and‐treat approach can increase the screening intervals.^26^ It can be therefore considered as an attractive option to improve the cost‐effectiveness of screening particularly in resource‐constrained countries. Studies that compared the sensitivity of the conventional cytology tests to that of HPV test concluded that the latter may be more effective for the purpose of precancer and cancer screening. In our study, the sensitivity and specificity of *careHPV*™ test to detect CIN2+ lesions were, respectively, 80.8% and 68.5%. HPV‐16 genotyping was the less sensitive (25.2%) but as reported elsewhere,^27^ it turned out to be the most specific (92.2%) test to detect CIN2+ lesions. The performance of *careHPV*™ test has been evaluated in other settings among women in the general population. In a cross‐sectional study among women attending routine cervical cancer screening in Tanzania, the sensitivity and specificity of the *careHPV*™ test to detect high‐grade cervical lesions or cancer (HSIL+) were 88.9% and 78.9%, respectively.^28^ In a multi‐country study, cervical *careHPV*™ testing was the most sensitive and had the best NPV for CIN2+ and CIN3+ as compared to vaginal *careHPV*™ testing, visual inspection with acetic acid, or conventional pap smear.^29^ In nine cases of CIN2+, careHPV™ test was negative. This could be explained by inaccurate histological interpretation or poor sample quality. The accuracy of *careHPV*™ was higher than the conventional Pap smear but almost equivalent to Siriraj liquid‐based cytology. The relatively low accuracy of careHPV™ could result in more false‐positive results and over‐referral for colposcopy.

Given its clinical performance and affordable cost, *careHPV*™ test represents an attractive screening option for the screen‐treat approach recommended by the WHO. It could also replace the need for colposcopy examination which can be limited in developing countries due to the lack of expertise or equipment.

*careHPV*™ test has been evaluated in rural China in the general population of women aged 25–65 years of age and was shown to be comparable to the gold standard HPV test Hybrid Capture 2 (HC2).^30^ An excellent agreement was also found between these two HPV tests in a population of 149 women living with HIV‐1 in African countries.^31^ In our study, the positivity rate of *care*HPV™ was 41.9% and the sensitivity of the test to detect CIN2+ was 80.8%, almost similar to those of 93.3% and 94.3% observed in two studies performed among women living with HIV‐1 in Africa.^32^, ^33^ The specificity of *careHPV*™ in the present study (68.5%) was higher than that (58%) reported in the Segondy et al. study.^32^ The discrepancy in the observed specificity between the two studies cannot be explained by differences in HR‐HPV prevalence, since the rates were similar (41%) in the two studies. The overall rate of HR‐HPV positive in our samples was similar to what has been reported by another study carried out in Lao PDR.^34^

Women infected with HPV‐16 and/or HPV 18 are more likely develop CIN2 or worse lesions than those infected with other high‐risk genotypes.^35^ Genotyping for these strains alone or in combination with other screening methods could therefore result in a better identification of high‐risk women.

As in other parts of the world, epidemiological studies in Southeast Asia have shown that the distribution of cervical HPV genotypes differs across populations, and oncogenic genotypes other than HPV‐16 and HPV‐18 are frequent.^36^, ^37^ HPV‐16 followed by HPV 52 and 68 were the most common genotypes in our study. HPV‐16 and HPV 68 were also among the most common genotypes in healthy women in Lao PDR.^34^ Phongsavan et al. also reported genotypes 33/52/58 in 4.3% and 16 in 3.1%, of their samples in Laos.^19^ HPV 51 and 70 (5.0%), followed by HPV‐16 (4.6%), HPV 71 (4.1%), and HPV 81 (3.7%) were the most common types found among young female sex workers (15–29 years) in Cambodia.^38^ HPV‐16 (17.9%), HPV 90 (16.6%), and HPV7 1 (10.3%) were reported in Thailand among patients during routine check‐up or investigation and treatment in hospital.^36^ In Vietnam, HPV 52 was the most common type (11.4%), followed by HPV‐16 (6.6%), and HPV 58, 62, and 51 (4.3% each).^37^

To further increase the screening rate, self‐collected vaginal samples were used in a study carried out in slums of Hyderabad.^26^ The study provided evidence of the feasibility and acceptance of the self‐collection.^26^ Another study carried out in rural areas of India compared the performance of *careHPV*™ on self‐collected vaginal samples or clinician‐collected cervical samples.^39^ The sensitivity of the test for detecting CIN2+ was 53.1% for cervical samples and 40.6% for vaginal samples. The loss of sensitivity should however to be balanced with the potential increase in screening coverage. The use of self‐collecting device for cervical cytology may have applications in Lao PDR. Self‐sampling has been reported as highly acceptable regardless of age, educational background, and residence in rural areas of Lao PDR.^40^

Cervical cancer screening requires a sensitive and specific test. However, the highly trained personnel and sophisticated laboratory equipment needed to achieve this level of performance are often lacking in developing countries. *careHPV*™, was designed specifically, and approved by the WHO, for application in low‐resource public health settings to screen women 30 years of age and older. The test procedure is simpler than other assays and has a faster time to results. Therefore, this test represents an attractive screening option in Lao PDR and similar countries. This is particularly true for women living with HIV‐1 because of the higher prevalence of chronic HPV infection in this population. The implementation of information campaigns and screening programs in HIV attending centers would be a significant step toward the set‐up of such programs at national level. This project was the opportunity to strengthen the skills of Laotian healthcare actors who received additional training on screening, diagnosis, and treatment of cervical cancer.

The results of LaoCol study can be used by local health authorities as a basis to improve their national cervical cancer screening policy and to establish HPV vaccination programs.

## Supporting information

Supplementary Material

## Data Availability

The data that support the findings of this study are available on request from the corresponding author.
