# Supplementary material for: Efficacy of careHPV™ human papillomavirus screening versus conventional cytology tests for the detection of precancerous and cancerous cervical lesions among women living with HIV‐1 in Lao People's Democratic Republic
Source: Cancer Med. 2022 Mar 8;11(9):1984–94. doi: 10.1002/cam4.4502 (PMC9089224; doi:10.1002/cam4.4502)
Supplement: Supplementary file 1 — Supplementary Material [file CAM4-11-1984-s001.docx]

**Supplementary material**

**Figure S1:** Study flow chart

**GYNECOLOGIST – PRIMARY VISIT**:
Gynecological examination, intervention + specimen collection

(n=644)

**Cervical sample specimens**

(n=643)

**Colposcopy & + /- Biopsy*** (*if anomaly found during the colposcopy)

(n=643)

**+/- Biopsy specimen**

**(n=631)**

**PAPs
sample**

**Genotyping sample**

**careHPV
sample**

**Signed informed consent**

(n=772)

Specimen conditioning, shipment to the CICML and laboratory processing laboratories

(n=631)

**Figure S2:** Description of tests and biopsy results among the study population

HPV: Human papillomavirus; HR: High-grade; LR: Low-grade; ASCUS-H: Atypical Squamous Cell evocating High grade lesion; LSIL: Low grade Squamous Intraepithelial Lesion; ASCUS-L: Atypical Squamous Cell evocating Low grade lesion; CIN: Cervical Intraepithelial Neoplasia; IC: Invasive Cancer**Table S1.** Sensitivity and specificity of HPV testing, conventional pap smear or Siriraj liquid based cytology to detect CIN2 in women living with HIV-1 stratified by age

| **Age group** | **N** | | **N_CIN2** | **Sensitivity** | | | **Specificity** | | |
| --- | --- | --- | --- | --- | --- | --- | --- | --- | --- |
| **careHPV™** | | | | | | | | | |
| 18-39 | 399 | | 30 | 80.0 (61.4-92.3) | | | 65.3 (60.2-70.2) | | |
| 40-49 | 151 | | 13 | 76.9 (46.2-94.9) | | | 74.6 (66.5-81.6) | | |
| ≥50 | 81 | | 4 | 100 (39.7-100) | | | 72.7 (61.4-82.3) | | |
| Total | 631 | | 47 | 80.8 (67.4-89.5) | | | 68.5 (64.6-72.1) | | |
|  | |  | |  | **HPV16** |  | |  |  |
| 18-39 | 399 | | 30 | 33.3 (17.3-52.8) | | | 90.8 (87.4-93.5) | | |
| 40-49 | 151 | | 13 | 15.4 (1.9-45.4) | | | 95.6 (90.8-98.4) | | |
| ≥50 | 81 | | 4 | 0.0 (0.0-60.2) | | | 93.5(85.5-97.9) | | |
| Total | 631 | | 47 | 25.2 (15.2-39.5) | | | 92.2 (89.8-94.2) | | |
| **Conventional Pap smear** | | | | | | | | |  |
| 18-39 | 399 | | 30 | 66.7 (47.2-82.7) | | | 65.0 (59.9-69.9) | | |
| 40-49 | 151 | | 13 | 61.5 (31.6-86.1) | | | 68.1 (59.6-75.8) | | |
| ≥50 | 81 | | 4 | 25.0(0.63-80.6) | | | 63.6 (51.9-74.3) | | |
| Total | 631 | | 47 | 61.7 (47.4-74.2) | | | 65.5 (61.6-69.3) | | |
| **Siriraj liquid based cytology** | | | | | | | | |  |
| 18-39 | 399 | | 30 | 80.0 (61.4-92.3) | | | 69.1(64.1-73.8) | | |
| 40-49 | 151 | | 13 | 69.2 (38.8-90.9) | | | 69.6 (61.2-77.1) | | |
| ≥50 | 81 | | 4 | 100 (39.8-100) | | | 68.8 (57.3-78.9) | | |
| Total | 631 | | 47 | 78.7 (65.0-88.0) | | | 69.2 (65.5-73.0) | | |

**Table S2.** Discordance and concordance for 14 HR-HPV of the *Care*HPV assay and the Papillocheck HPV assay in 591 pairs of samples with valid result

| Papillocheck* HPV | Negative test | Positive test | Total |
| --- | --- | --- | --- |
|  |  |  |  |
| *Care*HPV |  |  |  |
| Negative test | 311 | 69 | 380 |
| Positive test | 15 | 196 | 211 |
|  | N = 326 | N = 265 | N = 591 |
